# *Ixodiphagus hookeri* wasps (Hymenoptera: Encyrtidae) in two sympatric tick species *Ixodes ricinus* and *Haemaphysalis concinna* (Ixodida: Ixodidae) in the Slovak Karst (Slovakia): ecological and biological considerations

**DOI:** 10.1038/s41598-021-90871-7

**Published:** 2021-05-28

**Authors:** Alicja Buczek, Weronika Buczek, Katarzyna Bartosik, Joanna Kulisz, Michał Stanko

**Affiliations:** 1 Chair and Department of Biology and Parasitology, Medical University of Lublin, Radziwiłłowska 11 St., 20-080 Lublin, Poland; 2 Institute of Parasitology and Institute of Zoology, SAS, Hlinkova 3, 04001 Kosice, Slovakia

**Keywords:** Ecology, Environmental sciences, Natural hazards

## Abstract

*Ixodiphagus* endoparasitoid wasps are natural tick enemies that can reduce their abundance. In this study, we investigated the presence of *Ixodiphagus hookeri* in *Haemaphysalis concinna* and *Ixodes ricinus* ticks in the Slovak Karst (southern Slovakia) and analysed the ecological and physiological relationships in the parasitoid-host system. Unfed *H. concinna* and *I. ricinus* nymphs harvested from vegetation were fed on rabbits. The engorged specimens were kept at 25 °C and 75% RH until the emergence of the adult wasps. For the first time in Europe, we found the presence of *I. hookeri* in two species of ticks collected in the same locality and compared their development in these tick species. The prevalence of wasps in *H. concinna* and *I. ricinus* during their spring activity was estimated at 10.64% and 27.78%, respectively. The presence of the wasps did not affect the duration of nymph feeding. Engorged wasp-infected ticks achieved higher body mass than non-infected specimens. In both tick species, there were no differences in the length of the development period and the number and sex ratio of adult *I. hookeri*. The analysed indicators and characteristics of the *I. hookeri* wasp-tick system can be used in research on tick control.

## Introduction

The increase in the number of tick-borne diseases in humans and animals noted worldwide over the last few decades has aroused considerable interest in the chemical and biological methods for limitation of the number of their vectors, i.e. ticks, and in elucidation of the mode of the spread and persistence of tick-borne pathogens in the environment.

The use of biological agents, e.g. parasitoid *Ixodiphagus* wasps, for tick control was supposed to be an alternative to acaricides, as numerous studies reported emerging resistance of some tick populations to chemical products^1–3^ and toxic effects of acaricides on animals and humans^4–7^. Since no fully satisfactory elimination or replacement of chemicals with parasitoids in tick control has been achieved in large areas, it has been assumed that the presence of endoparasitoid wasps in tick habitats may reduce the abundance of these arthropods^8–11^.

As shown by investigations conducted so far, tick parasitoids may contribute to the circulation of pathogens in tick populations^12–14^ and contribute to the persistence of foci of tick-borne diseases with high importance for public health. Additionally, *Ixodiphagus* wasps transmit some commensal and symbiotic microorganisms that may play an essential role in tick biology and physiology, e.g. nutrition, development, reproduction, immunity, and adaptation to environmental conditions, and influence the occurrence and distribution of pathogens in ticks^13–19^.

Eight species of *Ixodiphagus* wasps were detected in ixodid ticks on different continents, e.g. in *Ixodes scapularis*, *Dermacentor variabilis*, and *Dermacentor andersoni* in North America^20–25^, *Amblyomma* sp. in South America^26^, *Hyalomma truncatum*, *Hyalomma rufipes*, and *Amblyomma variegatum* in Africa^27–29^, *Hyalomma anatolicum*, *Dermacentor silvarum*, and *Haemaphysalis concinna* in Asia^30,31^, *Ixodes holocyclus*, *Ixodes tasmani*, *Haemaphysalis bancrofti*, and *Haemaphysalis bremner* in Australia^32,33^, and *Ixodes uriae* and *Ixodes eudyptidis* in Oceania^34^. Endoparasitoid wasps were also found in *Rhipicephalus sanguineus* s. l. ticks colonizing large areas from North and South America through Africa to Asia^26,35–42^.

In Europe, representatives of the hymenopteran wasp *Ixodiphagus hookeri* were identified in several species of ticks, e.g. *Ixodes ricinus*^8,12–14,43–48^, *Ixodes persulcatus*^8,49^, *Dermacentor reticulatus* (= *Dermacentor pictus*)^8^, *Dermacentor marginatus*^50^, *Haemaphysalis concinna*^51–53^, and *R. sanguineus*^38^. Another endoparasitoid wasp species, i.e. *I. caucurtei*, was found in *I. ricinus* and *R. sanguineus*^54^.

Although *Ixodiphagus* wasps were already detected in several tick species, the knowledge of the determinants of their spread, behaviour, and development is still unsatisfactory. The ecological and trophic relationships between encyrtid wasps and their tick hosts have been poorly elucidated as well.

In the present study, we investigated the presence of *I. hookeri* wasps infesting ticks collected in a habitat in the Slovak Karst (southern Slovakia) and analysed environmental conditions that may promote this phenomenon. To determine the physiological relationships between hymenopteran wasps and their hosts, we examined the development of *I. hookeri* in nymphs of two species of ticks: *H. concinna* and *I. ricinus*.

## Results

The *H. concinna* and *I. ricinus* nymphs were collected in the Slovak Karst in a temperature range from 15.8 to 22.7°C and 47.8–89.9% humidity during their spring activity. The laboratory analyses showed that both tick species were infected by *I. hookeri* encyrtid wasps. In April, May, and June 2018, these parasitoids were detected in 25.0%, 5.0%, and 13.33% of *H. concinna* nymphs, respectively. Throughout the observation period, the prevalence of *I. hookeri* in the nymphs of this species was estimated at 10.64%. During the peak activity of *I. ricinus* nymphs in June, *I. hookeri* wasps were detected in as many as 27.78% of the tick specimens (Table 1).

**Table 1:** Occurrence of endoparasitoid *Ixodiphagus hookeri* wasps in *Haemaphysalis concinna* and *Ixodes ricinus* nymphs in the Slovak Karst, Slovakia.

Tick species	Collection date	T (°C)^a^	RH (%)^a^	Number of engorged nymphs studied	Number of engorged nymphs infected with parasitoids (%)	Number of *I. hookeri* parasitoids		
						Total in one nymph	Females	Males
*Haemaphysalis concinna*	19th April 2018	18.1	58.9	12 (25.0)	3 (25.0)	13	9	4
						7	3	4
						6	0	6
	29th May 2018	22.7	47.8	20 (5.0)	1 (5.0)	4	3	1
	27th June 2018	15.9	89.9	15 (13.0)	2 (13.0)	7	4	3
						8	6	2
Total	–	–	–	47 (10.64)	6 (10.64)	45	25	20
*Ixodes ricinus*	27th June 2018	15.9	89.9	18 (27.78)	5 (27.78)	14	6	8
						7	2	5
						17	8	9
						7	4	3
						14	10	4
Total	–	–	–	18 (27.78)	5 (27.78)	59	30	29

^a^Temperature (T) and relative humidity (RH) in the habitat during tick collection.

*Haemaphysalis concinna* and *I. ricinus* nymphs infected with *I. hookeri* and specimens without the endoparasitoid wasps kept in laboratory conditions at a temperature of 20°C ± 2°C and 50% ± 5% humidity fed on the rabbits for 4.0 ± 1.5 days and 4.4 ± 1.3 days as well as 4.2 ± 0.4 and 3.7 ± 0.6 days, respectively. The Mann–Whitney U test showed no statistically significant differences in the feeding length between the parasitoid-infected and non-infected nymphs of both tick species (Table 2).

**Table 2:** Comparison of the course of the parasitic phase of *Ixodiphagus hookeri*-infected and non-infected *Haemaphysalis concinna* and *Ixodes ricinus* nymphs.

Parameter	Tick species	Group of nymphs	N	M	SD	Min	Max	Statistical analysis	
								Test results	p value
Feeding period (FP, in days)	*H. concinna*	Non-infected	41	4.4	1.3	2	8	109.0 ^(1)^	0.6669
		Infected	6	4.0	1.5	2	6		
	*I. ricinus*	Non-infected	13	3.7	0.6	3	5	18.5 ^(1)^	0.1833
		Infected	5	4.2	0.4	4	5		
Unfed nymph body mass (UNBM, in mg)	*H. concinna*	Unfed	2^a^	0.244	0.034	0.220	0.268	–	–
	*I. ricinus*	Unfed	4^a^	0.162	0.027	0.138	0.191	–	–
Nymph engorgement mass (NEM, in mg)	*H. concinna*	Non-infected	41	5.12	0.81	3.67	7.62	t = 1.96; df = 5.50 ^(2)^	0.1014
		Infected	6	6.27	1.40	4.25	7.98		
	*I. ricinus*	Non-infected	13	3.87	0.80	2.62	5.22	t = − 3.003; df = 4.54 ^(2)^	0.0341
		Infected	5	6.53	1.92	4.16	8.69		
Increased nymph body mass (INBM)	*H. concinna*	Non-infected	41	21.0	3.3	15.0	31.2	t = − 1.96; df = 5.50 ^(2)^	0.1014
		Infected	6	25.7	5.7	17.4	32.7		
	*I. ricinus*	Non-infected	13	23.8	4.9	16.1	32.2	t = − 3.00; df = 4.54 ^(2)^	0.0341
		Infected	5	40.2	11.9	25.6	53.6		
Nymph feeding efficacy index (NFEI, in mg/day)	*H. concinna*	Non-infected	41	1.27	0.41	0.62	2.28	t = − 1.51; df = 5.37 ^(3)^	0.1873
		Infected	6	1.78	0.82	1.00	3.21		
	*I. ricinus*	Non-infected	13	1.09	0.35	0.63	1.74	t = − 2.32; df = 16 ^(2)^	0.0340
		Infected	5	1.56	0.47	1.04	2.17		

^a^Number of pools of nymphs with 10 specimens in each involved in the study, ^1^Mann-Whitney U test, ^2^test T, ^3^Cochran-Cox test, N—number of nymphs involved in the study, M—mean, SD—standard deviation, Min—minimum, Max—maximum.

The body mass of the wasp-infected engorged nymphs and its increase were higher than the mass of the non-infected specimens in both tick species (Table 2). The mean body mass was 6.27 ± 1.40 mg in the group of the engorged *H. concinna* nymphs infested by the parasitoids and 6.53 ± 1.92 mg in the group of the *I. ricinus* nymphs. The Cochran-Cox test showed significant differences in the nymph engorgement mass between the infected and non-infected *I. ricinus* nymphs (df = 4.54; p = 0.0341).

The increase in nymph body mass in the engorged *H. concinna* and *I. ricinus* nymphs infected with *I. hookeri* wasps was 25.7 ± 5.7 and 40.2 ± 11.2, respectively. The Cochran-Cox test showed statistically significant differences in the INBM values between the infected and non-infected nymphs in *I. ricinus* (df = 4.54; 0.0341) (Table 2).

Similarly, the nymph feeding efficiency index in both tick species was higher in the case of the *I. hookeri*-infected nymphs vs. the non-infected specimens. The NFEI value of was 1.56 ± 0.47 mg/day in the endoparasitoid-infested *I. ricinus* nymphs and 1.78 ± 0.82 mg/day in the *H. concinna* nymphs (Table 2). The t-test showed a significant difference in the NFEI value between the infected and non-infected specimens only in the case of *Ixodes ricinus* (df = 16; p = 0.0340).

Adult *I. hookeri* emerged from the *H. concinna* and *I. ricinus* nymphs on average at days 35.0 ± 3.8 and 35.4 ± 2.9, respectively, after the onset of the feeding of hungry specimens on the host and at days 31.0 ± 5.1 and 31.2 ± 2.9, respectively, after cessation of feeding and detachment of the engorged specimens from the skin. The t-test showed no significant difference in the length of the development of the parasitoids in the *H. concinna* and *I. ricinus* nymphs (Table 3).

**Table 3:** Development of endoparasitoid *Ixodiphagus hookeri* wasps in *Haemaphysalis concinna* and *Ixodes ricinus* nymphs collected in the Slovak Karst, Slovakia.

Parameter	Tick species	N	M	SD	Min	Max	Statistical analysis	
							Test t results	p
Period between the onset of tick feeding and emergence of *I. hookeri* adult stages (days)	*H. concinna*	6	35.0	3.8	29	38	t = 0.19; df = 9	0.8511
	*I. ricinus*	5	35.4	2.9	32	40		
Period between the end of tick feeding and emergence of *I. hookeri* adult stages (days)	*H. concinna*	6	31.0	5.1	24	36	t = 0.08, df = 9	0.9405
	*I. ricinus*	5	31.2	2.9	28	36		
Number of parasitoids per 1 mg of body mass of engorged nymphs	*H. concinna*	6	1.261	0.546	0.501	2.028	t = 1.89, df = 9	0.0952
	*I. ricinus*	5	1.743	0.190	1.437	1.907		

*N* number of nymphs involved in the study, *M* mean, *SD* standard deviation, *Min* minimum, *Max* maximum.

The number of *I. hookeri* adults that emerged from the *I. ricinus* nymphs ranged from 7 to 17 specimens and from 4 to 13 specimens in the case of the *H. concinna* nymphs (Table 1). Females and males were identified among the *I. hookeri* adults emerging from the cuticle of a majority of nymphs of both species. In one *H. concinna* nymph collected in April, only male wasps developed.

**Figure 1:**
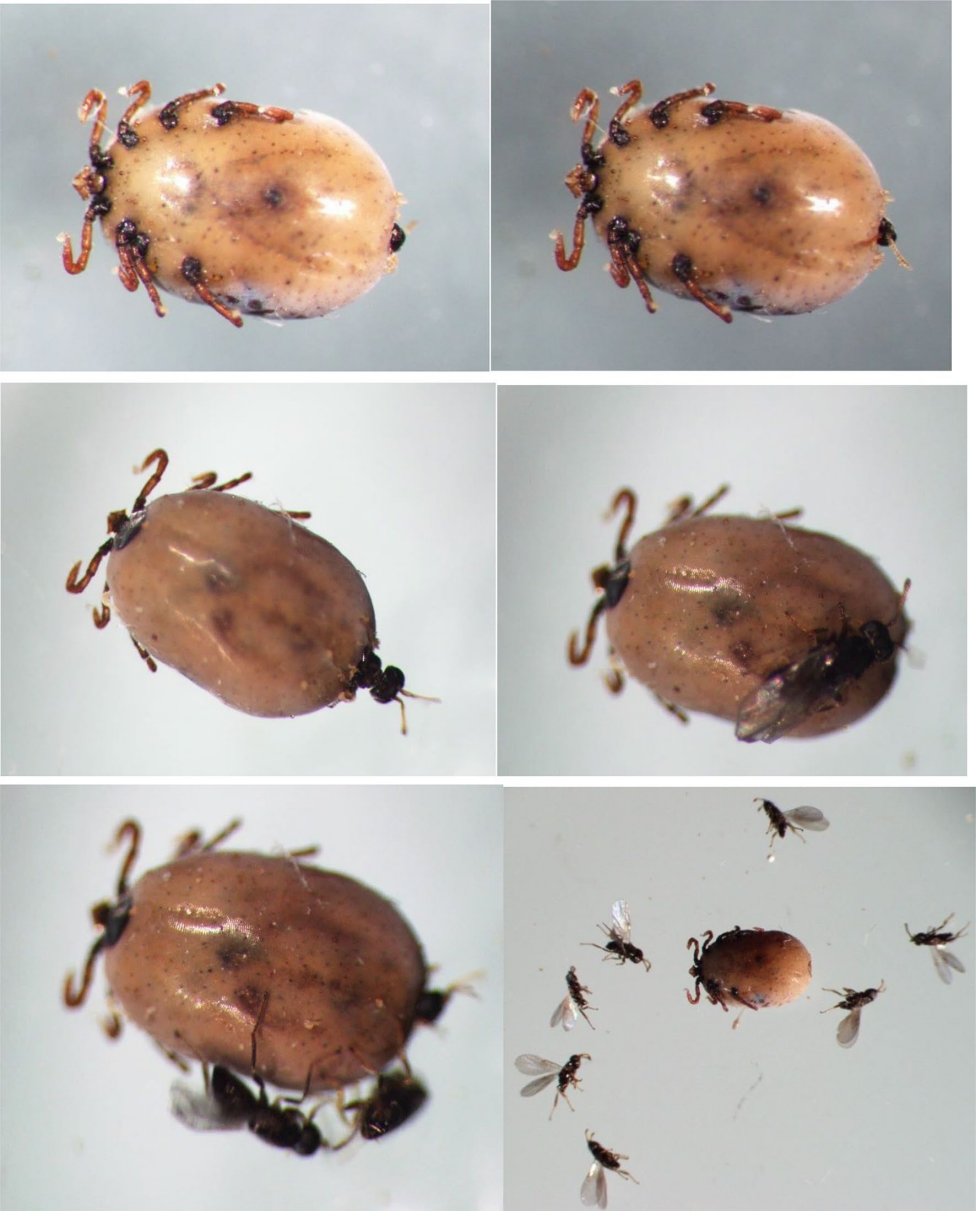
Emergence of endoparasitoid wasps *Ixodiphagus hookeri* from *Haemaphysalis concinna* nymph.

Before emergence from the tick cuticle, adult *I. hookeri* wasps pierced the back of their host's body with their mouthpieces. Our observations showed that the period between piercing the nymph cuticle and the emergence of the first wasp specimen was even up to 10 h long. After the emergence of the first adult wasp, the other specimens left the tick cuticle within a few seconds (Fig. 1).

**Figure 2:**
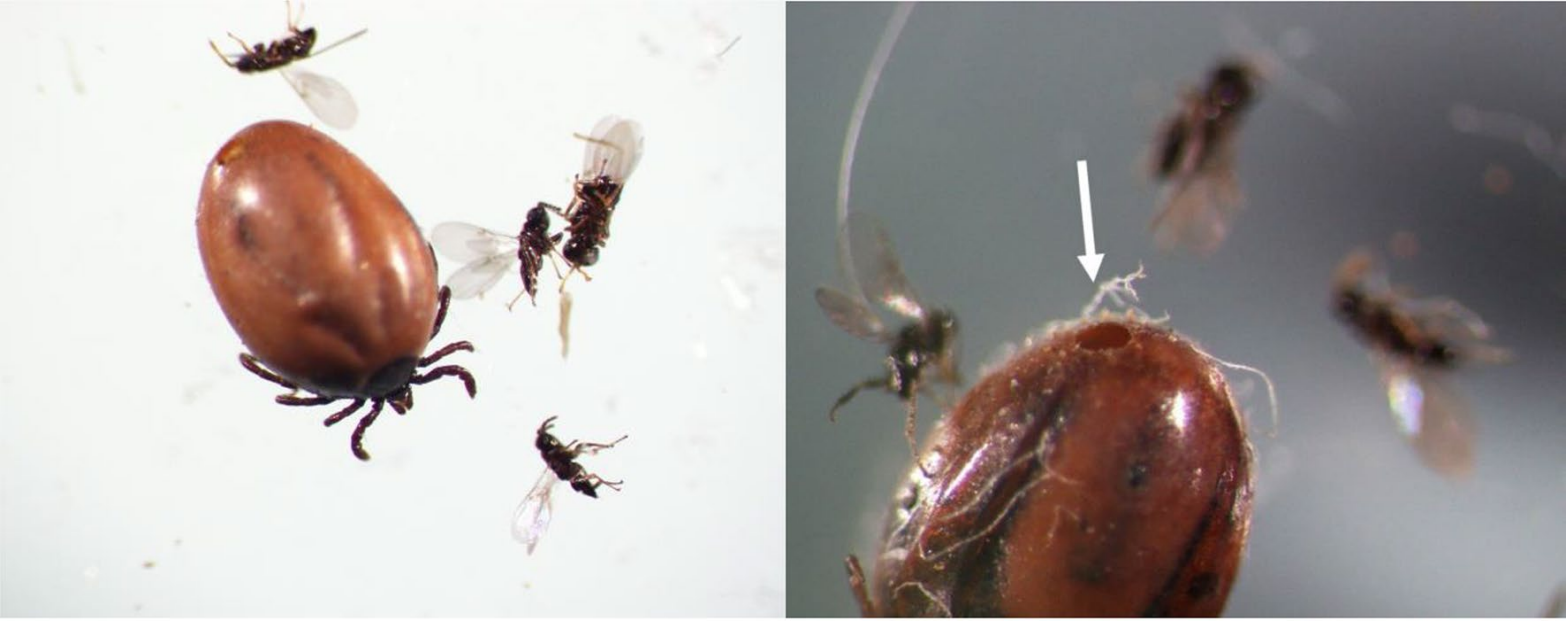
*Ixodes ricinus* nymph with endoparasitoid wasps *Ixodiphagus hookeri* (the arrow indicates the aperture in the tick cuticle through which the *I. hookeri* specimens emerged).

During development, *I. hookeri* wasps digested all internal organs of tick nymphs, leaving only the sclerotised cuticle (Figs. 1 and 2). The body of the wasp-infected nymphs changed colour and became matt. At the piercing site, a depression appeared on the posterior edge of the idiosoma due to the thinning of the cuticle. The adult wasps pierced an area near the posterior edge of the idiosoma in all nymphs (Figs. 1 and 2).

At the temperature of 25 °C and 75% RH, the adult *I. hookeri* wasps died after emergence through the chitinous body covers of *H. concinna* and *I. ricinus* nymphs within 3–6 and 2–8 days, respectively.

## Discussion

To the best of our knowledge, this is the first report on the presence of *I. hookeri* in *I. ricinus* and *H. concinna* nymphs from the same locality in Europe. The area near Hrhov (Slovak Karst) is a new locality of *I. hookeri* in Central Europe. In the area of former Czechoslovakia (now Slovakia and the Czech Republic), *I. hookeri* wasps were previously identified in *I. ricinus* in two other habitats: in Poteplí near Prague (50°05′16″N 14°25′16″E, Czech Republic)^55^ and near Bratislava (17°06′ E, 48°08′ N, SW Slovakia)^12,14^. The parasite was also detected in *H. concinna* in one locality near Šoporña (17°49’E, 48°15′N, SW Slovakia)^53^.

Besides the favourable geoclimatic conditions and the presence of numerous hosts of various tick developmental stages, the presence of the wasps in the Slovak Karst may be influenced by the species diversity of the tick hosts. The area is colonised by six tick species, i.e. *H. concinna, I. ricinus, Ixodes trianguliceps, D. reticulatus*, *D. marginatus*, and *Haemaphysalis inermis*^56^, which are characterised by allochronic or asychronic peak activity^57–66^. The different dynamics of the activity of tick larvae and nymphs present in the study area increases the opportunity for *I. hookeri* females to infest a host and lay eggs in its body.

During the study period from April to June and with the applied method for collecting ticks, we were not able to harvest juvenile stages (larvae and nymphs) of *D. marginatus*, *D. reticulatus*, and *I. trianguliceps* to determine whether these species present in the Slovak Karst locality are parasitized by encyrtid wasps as well.

Studies carried out to date indicate a correlation between the occurrence of *I. hookeri* wasps and the number of ticks and their hosts^24,48^. In forest habitats in the Netherlands, the relationship between the prevalence of *I. hookeri* in questing nymphs and the density of all developmental stages of *I. ricinus* and roe deer (*Capreolus capreolus*) was confirmed by Krawczyk et al.^48^. Similarly, Stafford et al.^24^ found that the degree of infestation of *I. scapularis* nymphs with these parasitoids in the USA was influenced by the density of ticks and white-tailed deer (*Odocoileus virginianus*). As suggested by these authors, the lower threshold limiting the incidence of wasps in *I. scapularis* is the density of 13–20 hosts per km^2^. It is still unclear whether the spread of endoparasitoid wasps in various regions is associated with the density of small mammals, i.e. hosts of tick larvae and nymphs. No relationship between the occurrence of endoparasitoid wasps and rodents was found in *I. ricinus* habitats in the northern part of Europe^48^.

As shown by Collatz et al.^67^, the questing behaviour of *I. hookeri* females is influenced by chemical substances emitted by tick-infested vertebrate animals. In turn, Takasu et al.^68^ confirmed experimentally that a hexane extract fraction from engorged nymphs stimulated ovipositor probing in *I. hookeri* females. Substances present in the faeces of engorged ticks may also be an attractant for female encyrtid wasps^69^. In Western Kenya, 30–45% of unfed *A. variegatum* nymphs and as many as 45–86% of engorged nymphs infesting cattle were infected by *I. hookeri*^10^.

The high percentage of *I. ricinus* tick nymphs (27.78%) infected with endoparasitoid wasps among the few randomly selected specimens collected in the Slovak Karst suggests their high prevalence in this habitat. Bohacsova et al.^14^ found *I. hookeri* in 13.8% of 50 *I. ricinus* nymphs collected in the area in Bratislava. In different research years, the number of *I. hookeri*-infected *I. ricinus* nymphs ranged in different years from 1.86% to 3.8% in Lower Saxony (northern Germany) and from 2.41 to 3.24% in the Baden-Württemberg region (south-west Germany)^44^. *I. hookeri* wasps were detected in 4–26% (average 9.5%) of *I. ricinus* nymphs in the south-west of the Netherlands in 2010^45^ and in 0.1–16% in the north-west of the country (Buunderkamp and Amsterdamse Waterleidingduinen) in 2019^48^.

A similar *I. hookeri* infection rate in *I. ricinus* nymphs was reported from the Mediterranean region. In France, *I. hookeri* parasitoids were found to infest from 3.2 to 12.5% of *I. ricinus* nymphs collected from roe deer in Chizé (Poitou–Charentes region in the west) and from 19.6 to 20% of nymphs collected from roe-deer and vegetation in Gardouch (Occitanie region in the south-west)^13^. In southern Italy, encyrtid wasps were detected in 8.2% of *I. ricinus* nymphs^46^. In turn, *I. hookeri* DNA was detected in only 0.4–2.3% of nymphs collected from plants in 2012–2014 in Finland^47^.

In the Slovak Karst locality, a high prevalence of *I. hookeri* parasitoid wasps was found in *H. concinna* nymphs throughout the spring activity period, with the highest value recorded for specimens collected in April. The literature does not provide information about the *I. hookeri* infection rate in *H. concinna* nymphs in other localities.

The length of the development of adult wasps and the short survival time of female wasps after emergence from the host body, also observed by other authors^9,11,13^, may indicate that the females of these insects laid eggs in the hungry *H. concinna* nymphs collected in April and May probably before the winter diapause of the ticks. The ability of non-embryonated *I. hookeri* eggs to survive during winter months inside hungry nymphs has been shown in *I. ricinus*^70^ and *I. scapularis*^25^. Another species of hymenopteran endoparasitoid wasps, i.e. *Ixodiphagus texanus*, can overwinter in engorged *D. variabilis* larvae and unfed nymphs^71^. In the *I. ricinus* and *H. concinna* nymphs collected in the Slovak Karst in June, *I. hookeri* eggs may have been laid before winter or during spring. It cannot be ruled out that the parasitoids infecting unfed or engorged larvae of these species were transferred transstadially to the nymphs, as observed in other tick species^21,70,72–75^.

The activity of *H. concinna* and *I. ricinus* larvae and nymphs in spring enables the ticks to find a host and ingest its blood, which is necessary to initiate the embryonic development of *I. hookeri*. In the Slovak Karst locality in spring, *Apodemus flavicollis, Apodemus agrarius, Crocidura leucodon, Microtus arvalis*, and *Myodes glareolus* were parasitized by *H. concinna* and *I. ricinus* nymphs and larvae^56^.

The presence of *I. hookeri* wasps in *H. concinna* and *I. ricinus* nymphs does not change the dynamics of tick feeding on the host but increases the body mass of engorged specimens and, consequently, elevates the values of indicators of the parasitic phase of the life cycle in comparison with these parameters in non-infected specimens. The body mass of the engorged *H. concinna* and *I. ricinus* nymphs, which was higher than 4.25 mg and 4.16 mg, respectively, may indicate *I. hookeri* infestation. As reported by Hu and Hyland^76^, the mean scutal index (ratio between body length and scutal length) in foraging *I. scapularis* nymphs increased with the increase in the diameter of *I. hookeri* eggs.

The embryonic development of wasps in *I. scapularis* nymphs ended 72 h after attachment to the host^76^. In the present study conducted at a temperature of 25 °C and 75% RH, the entire embryonic and post-embryonic development of *I. hookeri* adults in the ticks lasted approximately 35 days, and adult *I. hookeri* stages emerged approximately 31 days after the nymphs had finished foraging and detached from the skin of the host.

The body mass increase in the parasitoid-infected nymphs compared to that in the non-infected specimens was greater in the *I. ricinus* nymphs than in the *H. concinna* specimens. Regardless of the tick species, the number of wasp specimens per 1 mg of body mass of an engorged nymph was similar. The present investigations confirm that the development of *I. hookeri* is not dependent on the host species.

The determinants of the number of eggs produced by one *Ixodiphagus* wasp female in natural conditions and the number of eggs laid in one tick have not been elucidated to date. Experimental studies showed that an *I. hookeri* female laid 1.5 ± 0.25 and 28.6 ± 5.5 eggs in an engorged and hungry *A. variegatum* nymph, respectively^11^. It has been estimated that one *I. hookeri* female can lay approximately 120 eggs during a lifetime^77^, while an *I. texanus* female can lay even 200 eggs^21^.

In the present study, the number of *I. hookeri* adults emerging from one *H. concinna* and *I. ricinus* nymph was similar to the number reported for *I. ricinus* nymphs from a German and French population^13,44^ and nymphs of the North American species *I. scapularis*^15,23,78^. In turn, as many as 73 wasp specimens (on average approx. 20) were found to develop in *D. andersoni* and *D. variabilis* ticks^79^, and from 8 to 40 wasp adults were reported to develop in *A. variegatum*^75^. Hoogstraal et al.^80^ described a *Dermacentor auratus* nymph that died upon parasitism of 36 specimens of *Ixodiphagus mysorensis.*

In the present study, we observed a variable sex ratio of *I. hookeri* emerging from the *H. concinna* and *I. ricinus* nymphs; nevertheless, there were slightly higher numbers of females of these endoparasitoids than males throughout the study. Similarly, females dominated in the group of adult wasps infesting *I. ricinus* in a German and French population of this tick species^13,44^. The case of emergence of only *I. hookeri* males from the *H. concinna* nymph may indicate a possibility of parthenogenetic development of eggs in this species. Confirmation or rejection of this hypothesis will require further research.

The course of reproduction in hymenopteran endoparasitoid wasps has not been fully elucidated. It has been found that symbiotic bacteria *Wolbachia pipientis* can stimulate thelytokous parthenogenesis (development of females from unfertilised eggs) in unfertilised *Encarsia pergandiella* females (Hymenoptera: Aphelinidae)^81^. A similar effect on the reproduction of *Pnigalio soemius* (Hymenoptera: Eulophidae) is exerted by *Rickettsia* sp.^82^. As suggested by Logan et al.^71^, thelytokous or arrhenotokous reproduction (development of males from unfertilised eggs) may also occur in *Ixodiphagus* wasps in certain conditions, but the association of bacteria with the reproduction type in representatives of this genus has not been studied to date.

The abundance of *I. hookeri* in some Central European habitats in such tick species as *H. concinna* and *I. ricinus*, which are competent vectors of tick-borne diseases, prompts the need to investigate their biological role and participation in the transmission of pathogens in this region.

## Methods

### Study area

The locality in the Slovak Karst National Park near the village of Hrhov (48°34.899 N, 20°46.743E) is located at an altitude of 200–220 m a.s.l. at the foothills on the southern steep slopes of the Horný vrch Plateau. The area has a warm and moderately wet climate due to its location at the border of two types of climate: oceanic and continental.

The environmental conditions in the Slovak Karst support the richness of the flora of xerothermic species, calciphytes, and mountain dealpine and prealpine species. The plains are covered by oak-hornbeam forests, whereas oak forests occupy the hills and spruce forests grow on the karst pits. In turn, the northern parts are covered by beech forests. The fauna species are characteristic of the steppe and forest-steppe zones. There numerous animals inhabiting the study area are potential hosts of various developmental stages of ticks.

### Tick collection

Ticks were collected on the 19th of April (14 nymphs of *H. concinna*), the 29th of May (20 nymphs of *H. concinna*), and the 27th of June 2018 (17 nymphs of *H. concinna* and 33 nymphs of *I. ricinus*) in the early hours (between 9:00 and 11:00) for an hour each time. Unfed specimens were collected with the use of a 1-m^2^ white wool blanket swept over the vegetation along two 100-m-long transects. Ticks attached to the flag were transferred into transport containers. Concurrently, during each harvest, the temperature and humidity were measured in the habitat using electronic devices (Reed R6030 with an accuracy of 1 °C and 1% RH).

The species and developmental stage of the ticks were determined in laboratory conditions. All *H. concinna* and *I. ricinus* nymphs sampled in the field were used for investigation of thir development in laboratory conditions. Specimens that had ceased feeding were used in further analyses of their development.

### Laboratory analyses

Prior to the beginning of the experiments, unfed nymphs of both species were kept at room temperature for approximately 7–10 days. Immediately before feeding, nymphs in pools of 10 specimens were weighed using the RadWag WPA 120/C analytical balance with an accuracy of 10^–4^ g 0.001 g. This was the basis for the determination of the average body mass of one hungry nymph of each tick species.

Next, the unfed nymphs were transferred into canvas bags attached to the shaved skin of Albinotic New Zealand rabbits (*Oryctolagus cuniculus*) kept at 20 °C ± 2 °C and 50% ± 5% humidity. Ten nymphs representing only one species were placed on one rabbit. The course of nymph feeding was checked at the same time every day. After detachment from the rabbit skin, engorged nymphs were removed from the host, weighed on the analytical balance, and transferred into rearing chambers with a temperature of 25 °C and 75% humidity. The study on the development of endoparasitoid wasps involved 12, 20, and 15 engorged *H. concinna* nymphs selected randomly from the first, second, and third collection round, respectively, and 18 engorged *I. ricinus* nymphs from the third collection.

One chamber contained one engorged nymph. At the same time every day, the nymphs were viewed using the Olympus SZX16 stereoscopic microscope until adult encyrtid wasps emerged. Chambers where the wasps emerged were checked on the subsequent days until the insects died. The dead adult wasps and tick nymphs were preserved in 75% ethyl alcohol. The species and sex of the endoparasitoid wasps were determined based on the morphological features, as described by Gahan^38^.

Photographs documenting the development of the wasps were taken using an Olympus SZX16 stereo microscope with an attached DP26 camera.

Based on the laboratory studies, the parameters of the development of the *I. hookeri* adults in the *H. concinna* and *I. ricinus* nymphs were determined. These included the length of the feeding period of wasp-infested nymphs (FP- from the beginning to the end of feeding), the length of development of adult wasps (from the beginning of feeding of infected nymphs to the emergence of the parasitoids), the length of the period between the end of nymph feeding and the emergence of the wasps, and the body mass of engorged nymphs infected with the parasitoid wasps (nymph engorgement mass NEM).

The following parameters characterising the *I. hookeri*-infected nymphs and the development of these parasitoids in both tick species were determined:

1. Increased nymph body mass
  INBM = body mass of an engorged nymph/body mass of a hungry nymph.
2. Nymph feeding efficacy index in wasp-infected specimens
  NFEI (g/d) = NEM/FP, where NEM—nymph engorgement mass, FP—length of the feeding period of a wasp-infested nymph.
3. Number of endoparasitoid wasps per 1 mg of the body mass of an engorged nymph indicating the number of wasp specimens per 1 mg of nymph body mass after the end of feeding.

The same parameters and indicators were determined for nymphs that were not infected with *I. hookeri* wasps. They were used for comparison of the course of the parasitic and non-parasitic phases in the infected and uninfected nymphs.

Laboratory animals were provided with food and water ad libitum, and every effort was made to minimize their stress during the experiment. All experimental procedures described below were carried out in accordance with the standards of care and use of laboratory animals (Article 48 of the Act of January 15, 2015 on the protection of animals used for scientific or educational purposes, Polish Journal of Law Item 266 and Directive 2010/63/EU of the European Parliament and of the Council of 22 September 2010 on the protection of animals used for scientific purposes). The study was approved by the Local Ethical Committee for Animal Experiments at the University of Life Sciences in Lublin (approval no. 64/2018 from 16th April, 2018).

### Statistical analysis

Measurable variables were described with the use of basic parameters: arithmetic mean, standard deviation (SD), and minimum and maximum values (min. and max.). The significance of the differences in the measurable variables between the two groups was determined with the following tests:

- t test—in the case of the normal distribution of the measurable variable and homogeneity of variance.
- the Cochran-Cox test—in the case of the normal distribution of the measurable variable but no homogeneity of variance.
- the Mann–Whitney U test—in the case of non-normal distribution of the measurable variable.

A p value < 0.05 was considered statistically significant. Statistical calculations were performed using the STATISTICA 10 PL statistical package.
